# Vitamin D-dependent rickets (VDDR) type 1: case series of two siblings with a CYP27B1 mutation and review of the literature

**DOI:** 10.1590/2175-8239-JBN-2020-0001

**Published:** 2020-09-11

**Authors:** Rachita Singh Dhull, Reena Jain, Bobbity Deepthi, Hae II Cheong, Abhijeet Saha, Mohit Mehndiratta, Srikanta Basu

**Affiliations:** 1 Lady Hardinge Medical College and associated Kalawati Saran Children Hospital, Department of Pediatrics, Division of Pediatric Nephrology, New Delhi, India.; 2 Seoul National University Children’s Hospital, Department of Pediatrics, Seoul, Korea.; 3 Seoul National University College of Medicine, Kidney Research Institute, Medical Research Centre, Seoul, Korea.; 4 University College of Medical Sciences, Department of Biochemistry, New Delhi, India.

**Keywords:** Familial Hypophosphatemic Rickets, Children, 25-Hydroxyvitamin D3 1-alpha-Hydroxylase, Mutagenesis, Insertional, Raquitismo Hipofosfatêmico Familiar, Crianças, 25-Hidroxivitamina D3 1-alfa-Hidroxilase, Mutagênese Insercional

## Abstract

Two siblings presented with clinical and biochemical features of rickets, initially suspected as hypophosphatemic rickets. There was no improvement initially, hence the siblings were reinvestigated and later diagnosed as having vitamin D-dependent rickets (VDDR) type 1 due to a rare mutation in the CYP27B1 gene encoding the 1α-hydroxylase enzyme. Both siblings improved with calcitriol supplementation. The initial presentation of VDDR is often confusing and algorithmic evaluation helps in diagnosis. We also present a brief review of the literature, including genetics.

## Resumo

Dois irmãos apresentaram características clínicas e bioquímicas do raquitismo, com suspeita clínica inicial de raquitismo hipofosfatêmico. Não houve melhora no início, portanto os irmãos foram reavaliados e, posteriormente, diagnosticados com raquitismo dependente de vitamina D (VDDR) tipo 1 devido a uma rara mutação no gene CYP27B1, que codifica a enzima 1a-hidroxilase. Ambos os irmãos melhoraram com a suplementação de calcitriol. A apresentação inicial do VDDR geralmente é confusa e a avaliação algorítmica ajuda no diagnóstico. Também apresentamos uma breve revisão da literatura, incluindo genética.

## Introduction

Vitamin D-dependent rickets type 1 (VDDR type 1) is a rare cause of refractory rickets, caused by mutation in the *CYP27B1* gene. Children with mutations are normal at birth and present at around 6 months to 2 years of age with similar symptoms as nutritional vitamin D deficiency rickets. Common symptoms of the disease are delayed motor milestones, hypotonia, muscle weakness, and bone deformities, while some may present with hypocalcemic seizures in early infancy. Laboratory findings include hypocalcemia, hyperparathyroidism, low levels of 1,25(OH)_2_D_3_, and normal or high serum 25(OH)D_3_ with radiographic features of rickets 1.

## Case Presentation

**Sibling 1**. A 30-month-old male child presented with complaints slow growth, unable to stand, and bowing of legs. Six months prior to this presentation, he received vitamin D injection in another hospital, following which there was no improvement in symptoms. On physical examination there was hypotonia, frontal bossing, rachitic rosary, widening of wrist, and bilateral genu varum. Biochemical tests showed hypocalcaemia, hypophosphatemia, phosphaturia, and high serum ALP and PTH levels with normal vitamin D (Table 1). The possibility of renal tubular acidosis was ruled out in view of negative history of polyuria and normal pH in blood gas. Hypophosphatemic rickets was considered a possibility as fractional excretion of phosphate in urine was 48%, so he was started on phosphate, vitamin D, and calcium supplements, but there was no improvement. Further, differential diagnosis of VDDR type 1 was considered and the patient was started on calcitriol at 30 ng/kg/day along with phosphate supplementation (Joulie’s solution) at 2 mmols/kg/day. Both biochemical and clinical improvement was seen after starting treatment (Table 1).

**Table 1:** Parameters of the two siblings before and after treatment.

Parameters	Sibling 1		Sibling 2	
	Before Treatment	After Treatment (15 months)	Before Treatment	After Treatment (15 months)
Weight (kg)	7.88	12.7	6	10.24
Height (cm)	72	89.5	69	83.5
Total calcium (mg/dL)	9 .2	9.9	8.8	9.9
Serum phosphate (mg/dL)	1.8	4.99	2.1	5.86
Alkaline phosphatase (IU/L)	1122	293	1238	318
25-OH vitamin D (ng/mL)	35.3	40.43	34.9	35.57
1,25-OH Vitamin D* (pg/mL)	-	77.2	-	97.4
PTH (pg/mL)	330.2	79.1	670.7	38.9
TRP (pg/mL)	52%		74.1%	

PTH: Parathyroid Hormone; TRP: Tubular Reabsorption of Phosphate.

* 1,25-OH Vitamin D was done after treatment was started.

**Sibling 2**. This patient was the younger brother of case 1. He presented at the age of 15 months with similar complaints. On examination, he had wrist widening, large open anterior fontanelle, and bowed legs. Laboratory tests showed hypocalcemia, hypophosphatemia, phosphaturia, and raised serum ALP and PTH levels with normal vitamin D levels (Table 1). X-ray of bilateral wrists was suggestive of rickets. The patient was started on calcitriol at 30 ng/kg/day and phosphate supplements at 2 mmol/kg/day, which resulted in fast amelioration of both biochemical and clinical abnormalities. He was able to walk and his height increased at a speed of 10 cm/year. Treatment was adjusted in both patients as required according to the levels of serum calcium and phosphate, PTH, and urinary calcium/creatinine ratio during subsequent visits. The dose of calcitriol was gradually decreased to (20 ng/kg/day and 10 ng/kg/day) respectively in sibling 1 and 2 over few months of follow-up. Similarly, Joulie’s solution was given with a starting dose for low phosphate levels and was tapered and stopped during follow-up. Regular renal ultrasound was done on follow-up visits.

Gene sequence analysis showed a homozygous c.1294C>T (p.Arg432Cys) mutation in exon 8 of the *CYP27B1* gene in both siblings. This mutation was previously reported in a patient with VDDR type 1, is pathogenic and functional assay revealed that this missense mutant retains only 6.9% of 1α-hydroxylase activity of the wild type^2^. The parents were non-consanguineous and asymptomatic. Although, unfortunately, genetic testing of parents was unavailable, it is predicted that both parents were heterozygous for the mutation. There was no pathogenic mutation in *PHEX* and *FGF23*.

## Discussion

Vitamin D is an inactive pro-hormone and its activation requires sequential hydroxylation by cytochrome P-450 enzymes, resulting in formation of calcitriol or 1-alpha, 25-dihydroxyvitamin D_3_ (1,25(OH)_2_D_3_). 25-hydroxy vitamin D 1-α hydroxylase is the rate-limiting enzyme in activation of vitamin D, primarily expressed in the kidney. In VDDR type 1, due to blockage of this enzyme activity, there is normal or elevated 25(OH)D_3_ level, despite low or low-normal serum 1,25(OH)_2_D_3_, mimicking vitamin D deficiency clinically and radiologically^3^. In previous studies from India, VDDR type 1 as cause of refractory rickets has not been confirmed by genetic studies^4^^,^ 5. A differential diagnosis of VDDR type 1 should be done in patients not responding to vitamin D supplementation, once RTA is ruled out. In our patients, hereditary hypophosphatemic rickets was suspected initially in view of hypophosphatemia, phosphaturia, raised ALP, and normal vitamin D levels, but there was no improvement in the symptoms and serum ALP levels after starting vitamin D, phosphate, and calcium supplementation. Hence, a diagnosis of VDDR type 1 was considered, which was confirmed by identification of homozygous mutation in the *CYP27B1* gene.

The plausible mechanism for phosphaturia in our index cases is secondary hyperparathyroidism. Fraser *et al.* in their original study showed that there are high circulating levels of PTH in stage 2 of VDDR^6^. PTH is a potent hormone that acts on G protein coupled PTHR1 receptor expressed on renal proximal and distal tubular cells, and decreases the phosphate reabsorption, hence causing renal leak of phosphate. This hypothesis is further supported by the fact that hypophosphatemia and phosphaturia were corrected by restoration of serum calcium levels using calcitriol and thereby suppression of hyperparathyroidism.

The *CYP27B1* gene is located on chromosome 12q13.3, consisting of 17 helices, 6 β-strands, and 9 exons spanning 4859 bases^7^. Yamamoto *et al.* also identified amino acid residues whose point mutations cause conformational changes of protein, leading to absence of enzymatic activity. The amino acid R432, which was found to be mutated in our patients, is a part of the highly conserved ERR motif in helix K, and the ERR triad is proposed to form the conserved core folding in mitochondrial CYPs, so the mutation R432C may cause a significant disruption in the protein structure^8^.

To date, 71 different mutations in *CYP27B1* have been reported in association with phenotypes of VDDR type 1 from different ethnic groups and different countries^9^. The reported mutations include missense mutations (most common, Table 2), deletions, insertions, nonsense mutations, splicing mutations, and so on^1^^,^ 10 ^-^ 12. The mutation seen in our patients was homozygous but the same mutation was previously reported as compound heterozygous mutations in a Chinese patient^2^. Few cases have been reported with normal concentrations of 25(OH)D_3_ and 1,25(OH)_2_D_3_ and having homozygous missense mutations^12^, which were considered to cause conformational changes in protein, limiting the enzyme efficiency. This clinical heterogeneity suggests a genotype-phenotype relationship in this gene similar to other CYP genes. As our patient needed continuous supplementation of calcitriol, we believe that the mutation c.1294C>T (p.Arg432Cys) is probably associated with severe enzyme inactivity.

**Table 2:** Genotype phenotype correlation in different missense mutations of CYP27B1.

Genotype	Phenotype
*Q65H*	These mutations are associated with no enzymatic activity, leading to severe phenotypes.
*R107H*	
*P112 L*	
*G125E*	
*P143 L*	
*D164N*	
*S323Y*	
*R335P*	
*P382S*	
*R389C*	
*R389G*	
*R389H*	
*T409I*	
*R429P*	
*R453C*	
*V478G*	
*P497R*	
*G57V*	These mutations are associated with 5.6 to 12.1 % enzymatic activity, hence leading to moderate to severe phenotypes. However, clinical heterogeneity was found in patients having G57V mutation.
*G73W*	
*R459C*	
*L333F*	
*R432C*	
*R492W*	
*E189G*	Partial 1-hydroxylase activity ranging from 2.3 to 22%.
*E189K*	
*L343F*	
*T321R*	Show no enzymatic activity but phenotype reported is mild.
*G102E*	This mutation has 80% reduction in enzymatic activity leading to severe phenotype but some phenotypes were reported to have mild disease.

## Conclusion

Although a rare disorder, VDDR type 1 must be considered even in countries where vitamin D deficiency is common. Genetic analyses are beneficial for early diagnosis and further management of probable familial cases.

## Abbreviations

VDDR type 1: Vitamin D dependent rickets type 1
PHEX: phosphate regulating endopeptidase homolog X-linked
FGF23: Fibroblast growth factor 23
CYP27B1: Cytochrome P450 family 27 subfamily B member 1
